# Influence of *APOE* locus on poor prognosis of COVID-19

**DOI:** 10.1016/j.heliyon.2021.e07379

**Published:** 2021-06-23

**Authors:** Juliana Carla Gomes Rodrigues, Pablo Pinto, Luciana Pereira Colares Leitão, Lui Wallacy Morikawa Souza Vinagre, Natasha Monte, Marianne Rodrigues Fernandes, André Salim Khayat, Paulo Pimentel de Assumpção, Ney Pereira Carneiro dos Santos, Sidney Emanuel Batista dos Santos

**Affiliations:** a Núcleo de Pesquisas em Oncologia, Universidade Federal do Pará, Belém, Pará, Brazil; b Laboratório de Genética Humana e Médica, Instituto de Ciências Biológicas, Universidade Federal do Pará, Belém, Pará, Brazil

**Keywords:** SARS-CoV-2, COVID-19, Susceptibility, *APOE*, Lung

## Abstract

The COVID-19 pandemic has infected over 25 million of people worldwide, 5% of whom evolved to death and, among of the active cases, more than 60 thousand are classified as critical or severe. Recent studies revealed that ApoE, a protein encoded by *APOE* gene, may increase the risk of severe COVID-19 cases. ApoE has been involved with prevention of tissue damage and promotion of adaptative immune response in the lungs. This study investigated frequencies distribution of alleles that alter the ApoE expression in lung tissues to trace a profile of these variants and associate them to COVID-19 clinical outcomes. Data about APOE expression levels was obtained from the Genotype-Tissue Expression Project and the allele frequencies of *APOE* variants was acquired from the populations included in the phase 3 release of the 1000 Genomes Project. A total of 128 variants showed a significant impact on the *APOE* expression in lung tissues (p < 0.0001). Linkage Disequilibrium analysis revealed that 98 variants were closely grouped into seven distinct haplotype blocks, of which six were composed of variants that significantly decrease *APOE* gene expression in the lungs. Most of the haplotypes with higher impact on *APOE* expression showed greater frequencies in Europeans and lower in Africans, which implies that European populations might be more susceptible to SARS-CoV-2 infection. The present study indicates a potential genetic contribution of *APOE* expression-modifying variants in modulating the prognosis of COVID-19.

## 1 Introduction

Apolipoprotein E (ApoE) is encoded by the *APOE* gene on chromosome 19 and has three common alleles, ε2, ε3, and ε4, which presents an extensive genetic diversity across ethnic populations. These allele frequencies vary widely (ε2, 5%–10 %; ε3, 65%–70 %; and ε4, 15%–20 %) [1].

The function of ApoE is to play a pivotal role in the transport, metabolism, and homeostasis of cholesterol and other lipids by serving as a ligand for low-density lipoprotein receptors (LDLRs) and heparan sulfate proteoglycans (HSPGs) (Martínez-Martínez et al., 2020; Hatters et al., 2006) [2,3].

Polymorphisms in *APOE* gene have been extensively described as major risk determinants of late-onset Alzheimer disease and cardiovascular disorders; in both conditions, the *APOE∗ε4* allele confers an increased risk and the *APOE∗ε2* allele confers a decreased risk in comparison with the common *APOE∗ε3* allele [4, 5].

Additionally, compelling evidence has been demonstrating the ability of ApoE isoforms in modulating immune responses through both proinflammatory and anti-inflammatory cytokines, which is closely linked to a predisposition to autoimmune disorders and infections [6]. [7] Several studies have reported the association of ApoE isoforms with the life cycle, pathogenesis, and outcome of different viral infections, such as varicella-zoster and Epstein Barr viruses [8], herpes simplex virus [9], human immunodeficiency virus [10], hepatitis B virus [11], and hepatitis C virus [12].

Recently, a study conducted with the UK Biobank (UKB) community cohort, has revealed that ApoE, specifically the ε4∗ε4 genotype, increases the risk of severe Coronavirus disease 2019 (COVID-19), caused by the severe acute respiratory syndrome coronavirus 2 (SARS-CoV-2) [13]. This association was strongly significant, despite patients’ pre-existing comorbidities, such as dementia, cardiovascular disease, and type-2 diabetes.

Although apoE is mainly expressed in the liver, it is also expressed by alveolar macrophages, type I and type II alveolar epithelial cells, and pulmonary artery smooth cells in the lung (Yao et al. 2016; Kockx, Traini, and Kritharides 2018) [14,15]. So, a possible reason for the relation between COVID-19 severity and *APOE* variants is that *APOE∗ε4* might moderate macrophage pro-/anti-inflammatory phenotype in the lungs, which could drive to more extensive tissue damage.

**Figure 1:**
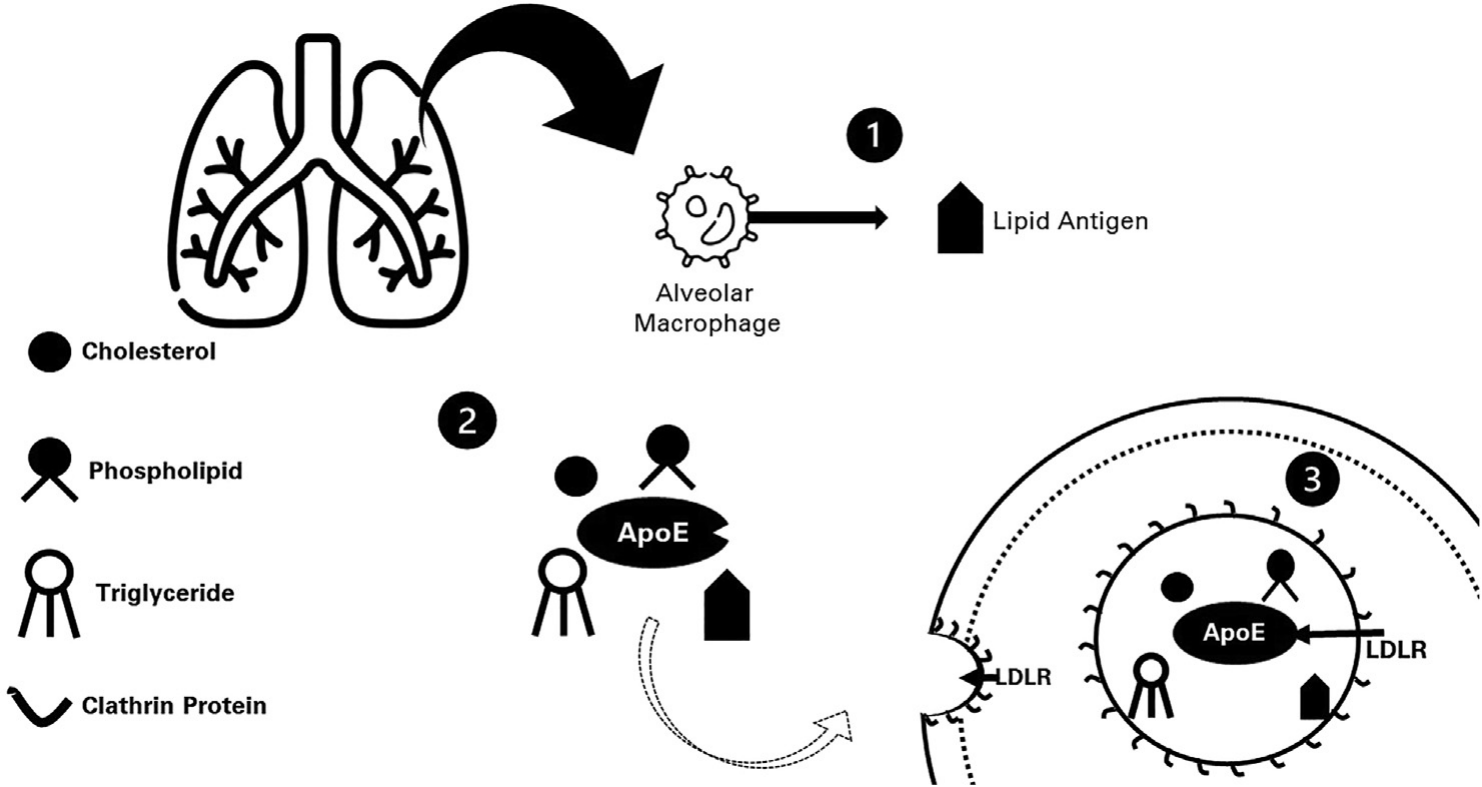
Modulation of lipid transport in the lung by apolipoprotein E (ApoE). (1) Exogenous pathway for apoE-mediated presentation of lipid antigens. ApoE facilitates the low-density lipoprotein receptors (LDLRs) internalization of mycobacterial lipid antigens for presentation by antigen-presenting cells; (2) ApoE mediates the cellular uptake of cholesterol, phospholipids, and triglycerides in lipoprotein particles, interacting with low-density lipoprotein receptors (LDLRs) located on the cell surface; (3) LDLRs are internalized in cells within the endocytic vesicles lined by clathrin proteins, which assist in the receptor-mediated endocytosis process. This image has been designed using resources from Flaticon.com.

ApoE has been demonstrated to be a key protein to maintain normal lung homeostasis. To exert this role, ApoE acts controlling hypercholesterolemia (Figure 1), which prevents systemic inflammation and oxidative stress that may lead to pathological lung and vascular remodeling changes [15]. Also, this protein still augments adaptive immunity and host defense in the lungs through the mediation of the LDLR-dependent internalization of mycobacterial lipid antigens by antigen-presenting cells [15].

The objective of this study is to investigate the frequency distribution of alleles responsible for altering gene expression of *APOE* in lung tissues to trace a profile of these variants and link them to poor clinical outcomes of COVID-19 patients worldwide. Since APOE expression has been linked to the prevention of tissue damage and promotion of adaptative immune response in the lungs, our analyses focused on the decreased expression of APOE in lung tissues.

## 2 Material and methods

All data concerning expression levels of *APOE* variants in lung tissues (means of expression, p-values, and expression quantitative trait loci [eQTL]) was obtained from the Genotype-Tissue Expression (GTEx) project (available at: https://www.gtexportal.org). The GTEx project is a centralized data source for information on inherited genetic variation and gene expression in 54 non-diseased tissue sites across nearly 1000 healthy individuals. Detailed information regarding the methodological procedures for the GTEx project have been previously described [16, 17].

Data regarding allele frequencies of the APOE variants evaluated here was from the populations included in the phase 3 release of the1000 Genomes Project (available at http://www.1000genomes.org) [18]. These data included populations of African (AFR), Admixed American (AMR), East Asian (EAS), European (EUR), and South Asian (SAS) descent. Linkage Disequilibrium (LD) was estimated and the haplotype blocks were compiled using the package “LDlinkR: An R Package for Calculating Linkage Disequilibrium” [19]. The cutoff for haplotype variants frequency was 0.5%. Details on haplotype blocks formation, such as the group of polymorphisms for each haplotype block, are shown in Supplementary Data.

The differences in gene expression among the haplotype blocks were evaluated by a one-way Analysis of Variance (ANOVA), and the significance of the difference between the most divergent blocks was determined by Tukey's HSD test. All analyses were run in RStudio v.3.5.1, using the “multcompView” package, 157 v.0.1–8 [20].

## 3 Results

A total of 128 variants has a significant impact on the *APOE* expression in lung tissues. These mutations are listed in Table 1, which also shows which allele decreases the gene expression and the distribution of its allelic frequencies in the Africa, America, East Asia, Europe, and South Asia populations of the 1000 Genomes Project Database (Table 1).

**Table 1:** Characteristics of variants that have a significant impact on the *APOE* expression in lung tissues and their allele distribution in world populations.

Variant Id	P-value	NES^a^	wt/wt	wt/var	var/var	Allele^b^	AFR	AMR	EAS	EUR	SAS
rs35136575	9.3e-10	0.27	-0.05833	0.08268	0.2858	C	0.1853	0.2017	0.1091	0.2376	0.2546
rs73045691	3.1e-17	0.31	-0.2055	0.05833	0.5972	G	0.326	0.4135	0.5526	0.329	0.3241
rs34810028	6.4e-12	0.24	-0.3728	0.05103	0.2503	A	0.6399	0.7161	0.7292	0.492	0.6288
rs12976395	3.2e-12	0.25	-0.4255	0.04860	0.2780	G	0.8011	0.7349	0.7272	0.492	0.6299
rs34041051	3.1e-18	0.32	-0.2104	0.05589	0.6383	T	0.329	0.415	0.5575	0.329	0.3272
rs35336243	3.1e-18	0.32	-0.2104	0.05589	0.6383	A	0.3268	0.415	0.5536	0.33	0.3231
rs12977604	4.6e-12	0.24	-0.3728	0.04860	0.2579	C	0.6399	0.7161	0.7282	0.492	0.6309
rs10413096	4.4e-10	0.23	-0.1684	0.02672	0.2907	G	0.6392	0.7161	0.7282	0.4911	0.6329
rs73045696	5.4e-18	0.32	-0.1685	0.02672	0.2907	G	0.3064	0.4121	0.5536	0.329	0.3231
rs59859410	5.5e-18	0.31	-0.2055	0.05103	0.6178	A	0.3132	0.4164	0.5536	0.331	0.3231
rs111809714	1.1e-18	0.31	-0.2055	0.05103	0.5972	T	0.3018	0.4179	0.5536	0.331	0.3221
rs111606839	9.1e-18	0.31	-0.2154	0.07050	0.5684	A	0.292	0.4135	0.5466	0.328	0.3221
rs112784534	9.1e-18	0.31	-0.2229	0.07780	0.5684	G	0.2905	0.4135	0.5536	0.331	0.3231
rs200024166	9.1e-18	0.31	-0.2154	0.07050	0.5684	TCTC	0.2905	0.4121	0.5526	0.331	0.3221
rs4803773	6.1e-12	0.24	-0.4016	0.05103	0.2907	A	0.7269	0.7248	0.7272	0.492	0.6329
rs12721111	1.3e-17	0.32	-0.2055	0.07050	0.5627	C	0.2458	0.4092	0.5536	0.331	0.3231
rs5157	5.7e-12	0.25	-0.4095	0.01457	0.3059	T	0.8011	0.7363	0.7272	0.4911	0.6329
rs1132899	3.2e-12	0.25	-0.4629	0.04373	0.2983	T	0.7307	0.7248	0.7262	0.498	0.6391
rs5167	3.5e-19	0.32	-0.2704	0.03887	0.5856	T	0.4569	0.4553	0.5724	0.3668	0.3415
rs2288912	7.6e-11	0.23	-0.3937	0.01214	0.3212	C	0.7632	0.7291	0.6746	0.4861	0.636
rs2288911	1.9e-10	0.22	-0.3911	0.009716	0.2983	T	0.7731	0.7349	0.6736	0.493	0.6411
rs9304644	2.2e-11	0.24	-0.3728	0.05833	0.2907	C	0.5719	0.5879	0.5923	0.4483	0.5941
rs9304646	6.7e-11	0.23	-0.3728	0.03401	0.3366	T	0.621	0.5994	0.5952	0.4483	0.5982
rs4803774	8.4e-11	0.23	-0.3572	0.02186	0.3573	A	0.6218	0.5994	0.5982	0.4493	0.6022
rs4803775	1.1e-10	0.23	-0.4016	0.002429	0.3572	T	0.8033	0.7349	0.6587	0.4901	0.6411
rs7256684	1.5e-10	0.23	-0.3728	0.02915	0.3573	A	0.6233	0.6023	0.5903	0.4513	0.5971
rs5120	1.1e-10	0.23	-0.4016	0.002429	0.3572	T	0.8033	0.7349	0.6558	0.4911	0.6421
rs4803776	4.1e-12	0.25	-0.3937	0.002429	0.4096	T	0.8018	0.7305	0.6558	0.4911	0.6431
rs150448996	6.6e-13	0.27	-0.1832	0.1315	0.5401	G	0.2466	0.3804	0.503	0.2903	0.2618
rs1130742	6.6e-13	0.27	-0.1832	0.1315	0.5401	C	0.2458	0.3804	0.503	0.2903	0.2618
rs7257468	1.9e-10	0.23	-0.3728	0.03887	0.3573	C	0.6044	0.5994	0.5883	0.4513	0.6012
rs7258345	1.9e-10	0.23	-0.3728	0.03887	0.3573	T	0.6059	0.5994	0.5883	0.4513	0.6012
rs7257476	1.9e-10	0.23	-0.3728	0.03887	0.3573	C	0.6059	0.5994	0.5883	0.4513	0.5992
rs12709889	4.7e-13	0.27	-0.1807	0.1291	0.5401	G	0.2315	0.3775	0.502	0.2903	0.2597
rs10423208	1.9e-10	0.23	-0.3728	0.03887	0.3573	G	0.6059	0.5994	0.5883	0.4513	0.5992
rs10402642	1.9e-10	0.23	-0.3728	0.03887	0.3573	G	0.6067	0.5994	0.5883	0.4513	0.5992
rs7246900	7.1e-11	0.23	-0.3728	0.05833	0.2907	G	0.562	0.5893	0.5863	0.4513	0.5951
rs11083752	1.9e-10	0.23	-0.3728	0.03887	0.3573	G	0.6059	0.5994	0.5883	0.4513	0.5992
rs7248162	1.9e-10	0.23	-0.3728	0.03887	0.3573	T	0.6059	0.5994	0.5883	0.4513	0.5992
rs7247227	7.1e-11	0.23	-0.3728	0.05833	0.2907	A	0.5635	0.5893	0.5863	0.4513	0.5971
rs7247551	1.1e-10	0.23	-0.4016	0.002429	0.3572	G	0.8056	0.7349	0.6558	0.4911	0.6442
rs892101	7.1e-11	0.23	-0.3728	0.05833	0.2907	G	0.5628	0.5893	0.5843	0.4513	0.5961
rs7251501	1.0e-10	0.23	-0.3885	0.04373	0.3573	C	0.6059	0.5994	0.5863	0.4513	0.5992
rs7251503	1.0e-10	0.23	-0.3885	0.04373	0.3573	C	0.6059	0.5994	0.5863	0.4513	0.5992
rs12460346	1.9e-10	0.23	-0.3728	0.03887	0.3573	C	0.6067	0.5994	0.5863	0.4513	0.5992
rs12460347	1.9e-10	0.23	-0.3728	0.03887	0.3573	C	0.6067	0.5994	0.5863	0.4513	0.5992
rs7254723	1.9e-10	0.23	-0.3728	0.03887	0.3573	A	0.5991	0.5994	0.5863	0.4503	0.5971
rs12460352	1.9e-10	0.23	-0.3728	0.03887	0.3573	C	0.6067	0.5994	0.5863	0.4513	0.5992
rs112757114	1.9e-10	0.23	-0.3728	0.03887	0.3573	TA	0.6059	0.5994	0.5863	0.4513	0.5992
rs139764322	1.9e-10	0.23	-0.3728	0.03887	0.3573	T	0.6059	0.5994	0.5863	0.4513	0.5992
rs4803777	7.1e-11	0.23	-0.3728	0.05833	0.2907	G	0.5628	0.5893	0.5843	0.4513	0.5961
rs4803778	1.0e-10	0.23	-0.3469	0.02915	0.3780	T	0.6059	0.5994	0.5863	0.4503	0.5992
rs4803779	1.9e-10	0.23	-0.3728	0.03887	0.3573	T	0.6059	0.5994	0.5863	0.4513	0.5992
rs4803780	1.9e-10	0.23	-0.3728	0.03887	0.3573	C	0.6059	0.5994	0.5863	0.4513	0.5992
rs3760625	1.9e-10	0.23	-0.3728	0.03887	0.3573	G	0.6059	0.5994	0.5863	0.4513	0.5992
rs3760626	1.9e-10	0.23	-0.3728	0.03887	0.3573	A	0.6067	0.5994	0.5863	0.4513	0.5992
rs3760627	7.1e-11	0.23	-0.3728	0.05833	0.2907	T	0.5628	0.5893	0.5843	0.4513	0.5961
rs3760628	1.9e-10	0.23	-0.3728	0.03887	0.3573	G	0.6059	0.5994	0.5863	0.4513	0.5992
rs66867801	5.7e-11	0.23	-0.3728	0.05346	0.3136	C	0.5651	0.5893	0.5843	0.4543	0.5961
rs7259679	1.0e-10	0.23	-0.3885	0.04373	0.3573	C	0.6067	0.5994	0.5863	0.4543	0.6002
rs66771331	7.1e-11	0.23	-0.3728	0.05833	0.2907	G	0.5635	0.5893	0.5833	0.4543	0.5961
rs73047643	1.3e-12	0.27	-0.1832	0.1315	0.5401	T	0.2466	0.3818	0.503	0.2952	0.274
rs7245611	1.6e-10	0.23	-0.3728	0.02915	0.3573	T	0.6059	0.5994	0.5863	0.4513	0.5992
rs3760629	2.2e-10	0.22	-0.3728	0.03401	0.3780	A	0.6104	0.6009	0.5873	0.4513	0.6002
rs2238682	2.0e-12	0.27	-0.1832	0.1315	0.5401	C	0.2345	0.3746	0.502	0.2913	0.2607
rs7252480	2.0e-12	0.27	-0.1832	0.1315	0.5401	C	0.236	0.3746	0.502	0.2913	0.2607
rs4803781	1.3e-10	0.23	-0.3728	0.03887	0.3859	G	0.6112	0.6023	0.5873	0.4533	0.6002
rs2239375	9.4e-11	0.23	-0.3885	0.03887	0.3859	T	0.6112	0.6023	0.5873	0.4533	0.5992
rs204905	1.3e-10	0.23	-0.4016	-0.004858	0.3676	G	0.8048	0.7349	0.6567	0.4911	0.6431
rs1882752	3.1e-12	0.26	-0.1906	0.1389	0.5457	G	0.2678	0.3775	0.503	0.2992	0.2638
rs35566032	0.0000014	0.17	-0.1610	0.03887	0.1586	GA	0.3411	0.5548	0.5585	0.4125	0.5143
rs8100120	2.1e-10	0.22	-0.3572	0.03401	0.3573	T	0.6051	0.6023	0.5893	0.4523	0.6033
rs8100236	2.1e-10	0.22	-0.3572	0.03401	0.3573	T	0.6051	0.6023	0.5873	0.4523	0.5951
rs111997200	2.1e-10	0.22	-0.3572	0.03401	0.3573	T	0.6051	0.6023	0.5873	0.4523	0.5951
rs147901416	4.4e-10	0.31	-0.07780	0.1463	0.5457	C	0.0605	0.1859	0.2659	0.171	0.1789
rs112704499	4.3e-12	0.26	-0.1807	0.1242	0.5599	G	0.2315	0.3761	0.502	0.3002	0.2638
rs142534985	4.3e-12	0.26	-0.1807	0.1242	0.5599	TAACTC	0.2315	0.3761	0.502	0.3002	0.2638
rs10416261	0.0000019	0.17	-0.1635	0.03887	0.2005	C	0.3359	0.5519	0.5585	0.4036	0.5143
rs113073220	1.3e-10	0.23	-0.3572	0.03401	0.3859	A	0.6021	0.5965	0.5863	0.4473	0.5951
rs11878597	1.4e-10	0.23	-0.3572	0.02915	0.3937	T	0.6112	0.6009	0.5873	0.4433	0.5961
rs35193317	3.5e-10	-0.22	0.3366	-0.004858	-0.3937	T	0.1959	0.2608	0.3433	0.501	0.3599
rs4803783	1.4e-10	-0.23	0.5345	0.1266	-0.2503	C	0.4811	0.4712	0.4266	0.6531	0.6881
rs2142074	3.1e-12	0.26	-0.1857	0.1389	0.5599	A	0.236	0.3746	0.502	0.2913	0.2638
rs141408713	7.1e-12	-0.23	0.5261	0.05589	-0.2503	A	0.329	0.4553	0.4117	0.6471	0.6524
rs11668758	3.1e-12	0.26	-0.1857	0.1389	0.5599	C	0.236	0.3746	0.502	0.2913	0.2628
rs11665849	3.1e-12	0.26	-0.1857	0.1389	0.5599	T	0.236	0.3746	0.502	0.2913	0.2628
rs11669609	3.1e-12	0.26	-0.1857	0.1389	0.5599	C	0.236	0.3746	0.502	0.2913	0.2628
rs71352247	2.0e-10	-0.22	0.2907	-0.004858	-0.3885	T	0.1944	0.2666	0.3423	0.5119	0.3569
rs204482	6.4e-8	0.19	-0.1659	0.01214	0.1906	A	0.2693	0.5519	0.5863	0.4185	0.5644
rs57465754	5.9e-12	0.28	-0.1635	0.1413	0.5627	C	0.475	0.2579	0.4256	0.2256	0.2935
rs16979595	1.9e-8	0.25	-0.1071	0.2204	0.4361	G	0.233	0.2075	0.2679	0.1829	0.2106
rs2075620	5.2e-12	0.26	-0.1807	0.1266	0.5457	A	0.2345	0.3746	0.502	0.3012	0.2638
rs56803710	4.3e-12	0.26	-0.1807	0.1242	0.5599	C	0.236	0.3746	0.502	0.3012	0.2648
rs7253458	6.0e-12	0.26	-0.1832	0.1291	0.5457	C	0.236	0.3746	0.502	0.3012	0.2648
rs7257916	1.4e-10	-0.23	0.3366	0.002429	-0.4016	C	0.1959	0.2695	0.3433	0.5149	0.3599
rs8111069	1.7e-16	0.29	-0.2805	0.03401	0.5741	A	0.4781	0.4222	0.5308	0.341	0.3446
rs55762617	5.2e-12	0.26	-0.1807	0.1266	0.5457	C	0.236	0.3746	0.502	0.3012	0.2648
rs55778858	5.2e-12	0.26	-0.1807	0.1266	0.5457	G	0.236	0.3746	0.502	0.3012	0.2648
rs204474	6.9e-17	-0.29	0.5457	0.01943	-0.3263	T	0.2973	0.4452	0.3859	0.6302	0.6104
rs60876330	5.2e-12	0.26	-0.1807	0.1266	0.5457	C	0.236	0.3746	0.502	0.3012	0.2597
rs11083755	5.2e-12	0.26	-0.1807	0.1266	0.5457	C	0.236	0.3746	0.502	0.3012	0.2648
rs11672748	1.7e-16	0.29	-0.2805	0.03401	0.5741	A	0.4811	0.4222	0.5308	0.341	0.3456
rs3786505	3.6e-16	0.29	-0.2805	0.04373	0.5741	A	0.4788	0.4222	0.5288	0.341	0.3425
rs204468	2.2e-16	-0.29	0.5627	0.01457	-0.3366	C	0.2988	0.4452	0.3998	0.6262	0.6104
rs204467	1.4e-16	-0.29	0.5684	0.02915	-0.3469	G	0.2716	0.4438	0.3998	0.6262	0.6104
rs11669173	1.5e-10	-0.22	0.3366	0.002429	-0.3911	A	0.261	0.2738	0.3433	0.5149	0.3589
rs909134	2.8e-16	0.29	-0.2805	0.03887	0.5856	T	0.4849	0.4207	0.5308	0.341	0.3456
rs875255	0.0000013	-0.17	0.1291	0.07537	-0.2154	G	0.4221	0.3098	0.371	0.5447	0.4376
rs2075618	5.2e-12	0.26	-0.1807	0.1266	0.5457	G	0.2368	0.3746	0.502	0.3012	0.2648
rs2075619	1.3e-16	0.29	-0.2805	0.03887	0.5856	A	0.4856	0.4207	0.5308	0.341	0.3456
rs35080293	1.5e-11	0.25	-0.1857	0.1291	0.5401	T	0.2511	0.3804	0.502	0.3012	0.2648
rs57204168	4.3e-17	0.30	-0.2805	0.05833	0.5856	A	0.4372	0.4107	0.5288	0.341	0.3425
rs892131	7.2e-12	0.25	-0.1821	0.1291	0.5457	C	0.2458	0.3775	0.502	0.3002	0.2628
rs73047694	7.2e-12	0.25	-0.1832	0.1291	0.5457	G	0.2458	0.3775	0.502	0.3002	0.2648
rs56784978	7.2e-17	0.30	-0.2755	0.06319	0.5627	T	0.4274	0.4193	0.5298	0.34	0.3384
rs2376866	2.6e-10	0.31	-0.08024	0.1536	0.5457	C	0.059	0.1844	0.2669	0.171	0.1687
rs2376867	3.1e-11	0.23	-0.1906	0.08268	0.5345	T	0.4342	0.5187	0.5744	0.3489	0.3037
rs7251655	5.2e-12	0.26	-0.1807	0.1266	0.5457	C	0.2352	0.3761	0.502	0.3002	0.2577
rs35577563	5.2e-17	0.29	-0.3520	0.02915	0.5674	C	0.7277	0.5706	0.5982	0.3827	0.3814
rs35194062	5.7e-7	0.39	-0.01943	0.3469	-	C	0.0023	0.0346	0.001	0.0527	0.0031
rs34647054	1.7e-11	0.25	-0.2329	0.1463	0.5599	C	0.3979	0.4006	0.5069	0.3022	0.2597
rs34449594	0.0000055	-0.17	0.2005	-0.02429	-0.3885	C	0.1422	0.2666	0.256	0.4622	0.3742
rs2376868	0.000028	-0.16	0.1758	-0.02429	-0.3937	A	0.1452	0.2493	0.2589	0.4453	0.3497
rs12972222	0.000014	-0.16	0.1169	-0.01943	-0.2154	A	0.3101	0.2795	0.2679	0.4592	0.3742
rs150557566	0.000051	-0.15	0.1857	-0.04373	-0.3937	G	0.025	0.2363	0.2659	0.4443	0.3456
rs9749077	0.000051	-0.15	0.2154	-0.04373	-0.3963	G	0.0885	0.2464	0.2609	0.4533	0.3497
rs11668285	0.000080	-0.15	0.2154	-0.04373	-0.3728	A	0.0855	0.2464	0.2579	0.4533	0.3497
rs4803791	4.8e-10	0.26	-0.1659	0.1659	0.5457	G	0.4054	0.3386	0.4097	0.2575	0.3661

wt/wt: wild homozygote; wt/var: heterozygote; var/var: variant homozygote.

a Normalized effect size: defined as the slope of the linear regression and computed as the effect of the alternative allele (ALT) relative to the reference allele (REF) in the human genome reference.

b Allele that causes the most decrease in Apoe expression in lungs.

The Linkage Disequilibrium (LD) analysis was performed and revealed that 98 variants were closely grouped into seven distinct blocks (both D’ and r^2^ > 0.9). The blocks are divided into I-VII, of which six (I-VI) are composed of variants that significantly decrease *APOE* gene expression in the lungs (p < 10^−10^). The blocks are detailed in Table 2.

**Table 2:** LD blocks of variants that decrease APOE expression in lung tissues.

Haplotype Blocks^a^	Frequencies				
I	AFR	AMR	EAS	EUR	SAS
Wild type	0.6505	0.5749	0.4375	0.666	0.6646
Mutant	0.233	0.3991	0.5377	0.3241	0.3098
I.1	∗	∗	∗	∗	∗
I.2	∗	∗	∗	∗	∗
I.3	∗	∗	∗	∗	∗
I.4	∗	∗	∗	∗	∗
I.5	∗	∗	∗	∗	∗
I.6	∗	∗	∗	∗	∗
I.7	0.031	∗	∗	∗	∗
I.8	0.0204	∗	∗	∗	∗
I.9	0.0159	∗	∗	∗	∗
**II**	**AFR**	**AMR**	**EAS**	**EUR**	**SAS**
**Wild type**	0.1853	0.2594	0.2639	0.4831	0.34151
Mutant	0.59	0.7075	0.6677	0.4791	0.6155
II.1	0.1006	0.0101	∗	∗	∗
II.2	0.0439	0.0058	∗	∗	∗
II.3	0.0287	∗	0.0546	∗	∗
II.4	0.0136	∗	∗	∗	∗
II.5	0.0091	∗	∗	∗	∗
II.6	0.0068	∗	∗	∗	∗
**III**	**AFR**	**AMR**	**EAS**	**EUR**	**SAS**
**Wild type**	0.3737	0.3977	0.4008	0.5487	0.3937
Mutant	0.5514	0.5865	0.5804	0.4473	0.5859
III.1	0.0393	0.0086	∗	∗	∗
III.2	∗	∗	0.0089	∗	∗
III.3	0.0113	∗	∗	∗	∗
III.4	0.0076	∗	∗	∗	∗
III.5	0.0053	∗	∗	∗	∗
**IV**	**AFR**	**AMR**	**EAS**	**EUR**	**SAS**
**Wild type**	0.3843	3963	0.4107	0.5437	0.3875
Mutant	0.5552	5879	0.5823	0.4394	0.59
IV.1	∗	∗	∗	∗	∗
IV.2	∗	∗	∗	∗	0.0082
IV.3	0.0333	0.0058	∗	∗	∗
IV.4	0.0083	∗	∗	∗	∗
IV.5	∗	∗	∗	∗	∗
IV.6	∗	∗	∗	∗	∗
**V**	**AFR**	**AMR**	**EAS**	**EUR**	**SAS**
Wild type	∗	∗	∗	∗	∗
Mutant	∗	0.0058	∗	0.006	∗
V.1	0.8011	0.7233	0.6488	0.4821	0.6329
**V.2**	0.1914	0.2565	0.3323	0.495	0.3476
V.3	∗	∗	∗	∗	∗
V.4	∗	∗	0.006	∗	∗
V.5	∗	∗	∗	0.0089	∗
V.6	∗	∗	0.0089	∗	∗
**VI**	**AFR**	**AMR**	**EAS**	**EUR**	**SAS**
**Wild type**	0.6868	0.6023	0.4921	0.6928	0.7198
Mutant	0.2224	0.3718	0.499	0.2783	0.2434
VI.1	0.0121	∗	∗	0.005	0.0133
VI.2	∗	∗	∗	∗	0.0061
VI.3	∗	∗	∗	∗	0.0051
VI.4	∗	∗	∗	0.0099	∗
VI.5	∗	∗	∗	0.0089	∗
VI.6	∗	0.0058	∗	∗	∗
VI.7	0.0318	∗	∗	∗	∗
VI.8	0.0151	∗	∗	∗	∗
VI.9	0.0098	∗	∗	∗	∗
VI.10	∗	∗	∗	∗	∗
**VII**	**AFR**	**AMR**	**EAS**	**EUR**	**SAS**
**Wild type**	0.5628	0.6945	0.7252	0.5298	0.6176
Mutant	0.0242	0.232	0.2381	0.4423	0.3395
VII.1	∗	0.013	∗	0.0139	0.0245
VII.2	∗	∗	∗	0.005	∗
VII.3	∗	∗	0.0089	∗	∗
VII.4	∗	∗	0.0079	∗	∗
VII.5	∗	∗	0.0069	∗	∗
VII.6	0.2209	0.0303	∗	∗	∗
VII.7	0.1074	0.0144	∗	∗	∗
VII.8	∗	0.0058	∗	∗	∗
VII.9	0.0582	∗	∗	∗	∗
VII.10	0.0113	∗	∗	∗	∗
VII.11	0.0061	∗	∗	∗	∗

∗ Haplotype frequency is lower than 0.5%.

a Further details about haplotype blocks are available in the supplementary data.

Linkage disequilibrium block I is formed by the following variants (with the allele responsible for the decrease of *APOE* expression in the lungs: rs73045691 (G), rs34041051 (T), rs35336243 (A), rs73045696 (G), rs59859410 (A), rs111809714 (T), rs111606839 (DEL), rs112784534 (G), rs200024166 (INS), and rs12721111 (C). This block contains 11 haplotypes, however only two have frequencies in all populations (the wild-type, responsible for all the variants that decreases *APOE* expression, and the mutant type). Three haplotypes are exclusively observed in AFR populations, and six were not identified in any of the 1000 Genomes populations. Additionally, the wild-type haplotype has the highest frequencies (varying of 44%–66%), where the EAS population shows the lowest frequency (44%) and the EUR shows the highest (66%).

Linkage disequilibrium block II is composed of nine variants: rs34810028 (A), rs12976395 (G), rs12977604 (C), rs10413096 (G), rs4803773 (A), rs5157 (T), rs1132899 (T), rs2288912 (C), and rs2288911 (T). This block has eight haplotypes, all of them is observed in the AFR, four is seen in AMR, three have frequencies in EAS, and two are observed in both EUR and SAS populations. The wild type haplotype, which all variants are responsible for decreasing *APOE* expression in the lungs, is observed in all populations, with higher frequencies in the EUR (48%) and lower in the AFR (19%).

Linkage disequilibrium block III includes 20 variants: rs9304644 (C), rs9304646 (T), rs4803774 (A), rs7256684 (A), rs7257468 (C), rs7258345 (T), rs7257476 (C), rs10423208 (G), rs10402642 (G), rs7246900 (G), rs11083752 (G), rs7248162 (T), rs7247227 (A), rs892101 (G), rs7251501 (C), rs7251503 (C), rs12460346 (C), rs12460347 (C), rs7254723 (A), and rs12460352 (C). This block has seven haplotypes, in which only two (the wild-type and mutant) are observed in all populations. The AFR population has the greater variability, with frequencies of six haplotypes. The haplotype that has the most impact on *APOE* expression is also the wild type, which shows higher frequencies in Europeans (55%) and lower in Africans (37%).

Linkage disequilibrium block IV contains 21 variants: rs112757114 (INS), rs139764322 (T), rs4803777 (G), rs4803778 (T), rs4803779 (T), rs4803780 (C), rs3760625 (G), rs3760626 (A), rs3760627 (T), rs3760628 (G), rs66867801 (C), rs7259679 (C), rs66771331 (G), rs7245611 (T), rs3760629 (A), rs2239375 (T), rs8100120 (T), rs8100236 (T), rs111997200 (T), rs113073220 (DEL), and rs11878597 (T). This block has eight haplotypes, of which four are present in AFR, three in each AMR, and SAS, and two in EAS and EUR. Of all the haplotypes, only the wild-type and the mutant type are observed in all the populations. The most deleterious haplotype is the wild type, which significantly decrease *APOE* expression, and its higher and lower frequencies are also observed in EUR (54%) and AFR (38%), respectively.

Linkage disequilibrium block V is formed by seven variants: rs4803775 (T), rs5120 (T), rs4803776 (T), rs7247551 (G), rs204905 (G), rs35193317 (T), and rs71352247 (T). This block shows seven haplotypes, and the EAS and EUR populations have the greater variability, with frequencies of four of them. Differently of the other blocks, the AFR populations has the lower number of haplotypes of block V, only two of them. The haplotype that causes the larger reduction in *APOE* expression in the lungs is the V.2. This haplotype is identified in all populations, with higher frequencies in EUR (49,5%) and lower in AFR (19%).

Linkage disequilibrium block VI has the largest number of variants, 25: rs150448996 (DEL), rs1130742 (C), rs12709889 (G), rs73047643 (T), rs2238682 (C), rs7252480 (C), rs1882752 (G), rs112704499 (G), rs142534985 (INS), rs2142074 (A), rs11668758 (C), rs11665849 (T), rs11669609 (C), rs2075620 (A), rs56803710 (C), rs7253458 (C), rs55762617 (C), rs55778858 (G), rs60876330 (C), rs11083755 (C), rs2075618 (G), rs35080293 (T), rs892131 (C), rs73047694 (G), and rs7251655 (C). This block is composed of 12 haplotypes, albeit the allele frequency of 10 are rare and only two of them are observed in all populations, the wild and mutant types. The wild haplotype causes the greater reduction in APOE expression, and has the highest frequencies recorded, ranging from 49% in EAS to 72% in SAS.

Lastly, the block VII is formed by six variants: rs34449594 (DEL), rs2376868 (A), rs12972222 (A), rs150557566 (DEL), rs9749077 (G), and rs11668285 (A). This block shows 13 haplotypes, where the AFR has the greater variability (containing seven haplotypes) and SAS has the lesser variability (with frequencies of three haplotypes). The wild and mutant haplotypes are the only two that are observed in all populations. Here, the mutant haplotype causes the most impact on *APOE* expression, decreasing its gene expression in the lungs. This haplotype has a great difference of frequency among the populations, AFR (2%), AMR (23%), EAS (24%), EUR (44%), and SAS (34%). Once again, the Europeans has the highest frequencies while African has the lowest.

The pairwise comparison of the mean expression of each linkage disequilibrium blocks showed statistically difference (p < 2e-16). The blocks that are most distinguished in terms of decreased expression of APOE are blocks I and IV in relation to all blocks (p < 0.0001), except among themselves. A close analysis reveals that block V causes the most decrease in APOE expression in lung tissues, with a mean of 0.397, which can be observed in Table 3. Also, it is noteworthy that this block has extremely high frequencies in all evaluated populations (>98%).

**Table 3:** Pairwise comparison of the LD blocks. For each LD block, the sum of frequencies of haplotypes and the expression of *APOE* was computed concerning the mean of the median of normalized expression from eQTL results in GTEx portal.

Haplotype Blocks	Expression value	AFR	AMR	EAS	EUR	SAS
I	-0.207	0.767	0.601	0.462	0.676	0.690
II	-0.378	0.388	0.293	0.332	0.521	0.385
III	-0.374	0.449	0.414	0.420	0.553	0.414
IV	-0.369	0.445	0.412	0.418	0.561	0.396
V	-0.397	0.993	0.980	0.996	0.986	0.989
VI	-0.183	0.778	0.628	0.501	0.722	0.757
VII	-0.360	0.437	0.306	0.275	0.470	0.382
	$\bar{x}$	0.608	0.519	0.486	0.641	0.573

## 4 Discussion

The COVID-19 pandemic has infected over 25 million of today people worldwide, 5% of whom evolved to death, which is equivalent to more than 800 thousand of cases [21]. Still according to the Worldometer's COVID-19 data, of the actual active cases, more than 60 thousand are classified as critical or severe. Also, seven of the ten countries with the highest death rates per million are European (San Marino, Belgium, Andorra, Spain, United Kingdom, Italy, and Sweden).

To reduce disease burden and improve clinical outcomes of patients affected by COVID-19, it is fundamental to create monitoring strategies to identify those who are most susceptible to developing severe forms of the disease. One of the approaches consider interindividual genetic differences among patients. Following this premise, two large studies of patients with European-ancestry from the UK Biobank (UKB) cohort reported that individuals with *APOE* ε4∗ε4 genotype had an increase in the risk of developing severe COVID-19 and a four-fold increase in mortality after testing positive for COVID-19 [13,22].

The aim of this study was to shine new light on the influence of *APOE* genotypes in predicting poor prognosis of COVID-19, such as severity and mortality, through an examination of allelic frequencies of the variants that can alter patterns of this gene expression in lung tissues. We based our findings through publicly available data analysis of the GTEx platform and 1000 Genomes Project database.

The evaluation of the 98 expression-modifying variants of the *APOE* gene formed seven haplotype blocks with considerable levels of variation among the global populations. All the haplotype genotypes, except that from the VI block, that lead to the greatest reduction in APOE expression in lung tissues are more frequent in Europeans (with a mean of 0.507). Otherwise, their lowest frequencies are found in AFR (regarding II, III, IV, V, and VII haplotype blocks; mean of 0.231) and EAS populations (I and VI haplotype groups; mean of 0.465).

In vivo experiments models show that ApoE-deficient mice demonstrated greater increases in lung lavage protein levels, neutrophil counts, and cytokine expression than wild-type animals [23, 24]. These studies confirm the role of low levels of *APOE* expression in modulating inflammation in the setting of acute lung injury, which has been associated to an interleukin-6–dependent mechanism that increases endothelial cell permeability to oxidized LDL [25]. Interestingly, IL-6 is a major cytokine released during the “cytokine storm”, the main responsible for COVID-19 complications, primarily represented by the development of acute respiratory distress syndrome (ARDS) [26].

Together, these findings may explain why individuals of European-ancestry, who have higher frequencies of haplotypes that confer deficiency in *APOE* expression, may be at higher risk to COVID-19 severe course, as demonstrated by the current available epidemiology. Contrarily, individuals from Africa and East Asia regions, both carrying lower frequencies of expression-modifying haplotypes of *APOE,* have reported fewer numbers of severe cases and smaller rates of mortality of COVID-19.

Conflicting with our hypothesis, some investigations point ApoE as a causative for complications during COVID-19 clinical evolution. The authors sustained that individuals with ate least one copy of *APOE* ε4 alleles may be more prone to progress to severe illness from SARS-CoV-2 infection due to an exacerbated innate immune response followed by cytokine storm and resulting in ARDS (Moriarty et al. 2020; Goldstein et al. 2020) [27,28]. However, this cannot be proven by our results since the variants that composes the *APOE* ε4∗ε4 genotype are absent of the GTEx list of variants expressed in lung tissues.

ApoE's influence on the clinical aspects of COVID-19 may also be correlated with its intrinsic role in the pathophysiology of obesity. Aguilar and colleagues demonstrated that ApoE −/− mices fed with an obesogenic diet showed a significant increase in atherosclerosis and systemic inflammatory processes [29].Another study showed that the *APOE* Ɛ2 and Ɛ4 alleles were associated, respectively, with Body Mass Index (BMI) and a higher risk of atherosclerosis [30]. Both processes, atherosclerosis and systemic inflammation, predispose to the development of obesity, showing that variants and protein isoforms of the *APOE* gene have a direct impact on metabolic disorders.

Studies have already shown that obesity can be considered a risk factor for the severity and mortality of Sars-CoV-2 infections [31, 32]. A recent meta-analysis showed that inpatients who have a BMI greater than 30 demonstrate a poor prognosis and higher mortality and severity rates of COVID-19; the authors calculated that the risk for composite poor outcome increased by 5% for every 5 kg/mg^2^ increase in BMI [33].

The present study indicates a potential genetic contribution of *APOE* expression-modifying variants in assess poor prognosis of COVID-19, such as severity and mortality outcomes. These findings are in accordance with the higher incidence and mortality rates described in Europeans, who have greater frequencies of haplotypes that alter this gene expression in lungs. Future research is needed to describe these variants in countries that have become the epicenter of the pandemic, such as United States, India, and Brazil, and to validate our findings in cohorts of patients, better elucidating the real role of ApoE to COVID-19 biological mechanisms.

## Declarations

### Author contribution statement

Juliana Carla Gomes Rodrigues: Conceived and designed the experiments; Analyzed and interpreted the data; Wrote the paper.

Pablo Pinto: Analyzed and interpreted the data.

Luciana Pereira Colares Leitão, Lui Wallacy Morikawa Souza Vinagre, Natasha Monte: Analyzed and interpreted the data; Wrote the paper.

Marianne Rodrigues Fernandes, André Salim Khayat, Paulo Pimentel de Assumpção, Ney Pereira Carneiro dos Santos: Contributed reagents, materials, analysis tools or data.

Sidney Emanuel Batista dos Santos: Conceived and designed the experiments; Contributed reagents, materials, analysis tools or data.

### Funding statement

This work was supported by Fundação Amazônia de Amparo a Estudos e Pesquisa, Universidade Federal do Pará and Pró-reitoria de Pesquisa e Pós-graduação.

### Data availability statement

Data included in article/supplementary material/referenced in article.

### Declaration of interests statement

The authors declare no conflict of interest.

### Additional information

No additional information is available for this paper.

## Appendix A Supplementary data

Supplementary_data_V2 Supplementary_data_V2
